# N2E4, a Monoclonal Antibody Targeting Neuropilin-2, Inhibits Tumor Growth and Metastasis in Pancreatic Ductal Adenocarcinoma *via* Suppressing FAK/Erk/HIF-1α Signaling

**DOI:** 10.3389/fonc.2021.657008

**Published:** 2021-07-15

**Authors:** Li Wang, Lanlan Wang, Shengyu Wang, Zonglang Zhou, Zongjunlin Liu, Peilan Xu, Xian Luo, Ting Wu, Fanghong Luo, Jianghua Yan

**Affiliations:** ^1^ Cancer Research Center, Medical College, Xiamen University, Xiamen, China; ^2^ The 174th Clinical College of People’s Liberation Army, Anhui Medical University, Hefei, China

**Keywords:** neuropilin-2, monoclonal antibody, pancreatic ductal adenocarcinoma, metastasis, focal adhesion kinase

## Abstract

Pancreatic ductal adenocarcinoma (PDAC) is a highly aggressive malignancy with extremely limited treatment; the effective targeting strategy stays an urgent unmet need. Neuropilin-2 (NRP2), a multifunctional transmembrane non-tyrosine-kinase glycoprotein, enhances various signal transduction pathways to modulate cancer progression. However, the application value of NRP2 as a therapeutic target in pancreatic cancer is still unclear. Here, we detected the elevated NRP2 was associated with the poor prognosis of pancreas carcinoma. The mouse monoclonal antibody targeting NRP2 (N2E4) that could specifically bind to PDAC cells was developed. Moreover, N2E4 inhibits PDAC proliferation, migration, and invasion *in vitro*, and repressed growth and metastasis *in vivo*. Mechanistically, the effect of N2E4 was mainly related to the blocking of interaction between NRP2 with integrinβ1 to inhibit FAK/Erk/HIF-1a/VEGF signaling. Therefore, N2E4 has the potential for targeting therapy of PDAC. This study lays a foundation for the future development of NRP2-based targeted therapy for PDAC.

## Background

As the king of cancer, pancreatic ductal adenocarcinoma (PDAC) has the lowest 5-year survival rate of only 9% from 2009 to 2015 (1, 2). The survival benefit from surgical resection and chemotherapy for PDAC is extremely limited (3). In recent years, various molecularly targeted drugs have been used alone or in combination with chemotherapy drugs for PDAC treatment, including targeting angiogenic pathways, epidermal growth factor receptors (EGFR), cancer stem cells, and so on (4). However, little progress arises in the targeting treatment of PDAC compared to other cancers. Therefore, scientific trials to evaluate new targets for PDAC remain unmet medical needs.

Neuropilins (NRPs), transmembrane non-tyrosine-kinase glycoproteins, are the receptors of class-3 semaphorins, including two highly conserved homologous members, neuropilin-1 (NRP1) and neuropilin-2 (NRP2) (5). A great deal of researches shows that NRP2 is upregulated and associated with unfavorable prognosis in a variety of tumors (6, 7). As multifunctional membrane proteins, NRP2 enhances or modifies signal transduction pathways through interactions with various receptors or ligands to modulate cancer progression (8). For example, NRP2 interacts with and functions as a co-receptor for the α6β1 integrin in breast cancer cells, which facilitates α6β1 signaling and activates focal adhesion kinase (FAK) (9, 10). In particular, these studies demonstrate that NRPs can promote vascular endothelial growth factor (VEGF) signaling without VEGF receptor (VEGFR) involvement by co-opting integrin signaling (11). Furthermore, JJ Ou et al. found that the integrin/FAK/Erk pathway induces NRP2 activation, promoting tumor progression (12). The Erk cascades are not only mainly considered to be correlated with cancer cell proliferation, survival, and actin remodeling, but also upregulate the expression of hypoxia-inducible factor-1 (HIF-1) followed by VEGF (13). The upregulated VEGF then acts on the same cell *via* an autocrine manner to further enhance VEGF action (14).

According to Caunt, et al., blocking NRP2 function inhibited metastasis of breast cancer and glioma cells (15). Although not as intensely explored for functions of NRP-2 in PDAC, it is reliable that NRP-2 is involved in biological processes of pancreatic cancer such as cell survival and invasion (16). These findings suggest that NRP2 is a potential target for cancer therapy, but it is unclear whether PDAC therapy targeting NRP2 can improve tumor progression or cancer metastasis.

Given these findings, we have successfully obtained a monoclonal antibody targeting NRP2 (N2E4) that stably binds to the NRP2 b1b2 fragment by genetic engineering and hybridoma technology in the previous study (17). Large-scale production and purification (>95%) were established in our laboratory to guarantee to be sufficient for the study. In this work, we comprehensively assessed the expression of NRP2 in pancreatic cancer, identified the N2E4 target protein (NRP2) in PDAC cells, and further elucidated the positive therapeutic effect of N2E4 *in vitro* and *in vivo* and the underlying mechanisms. We provided evidence that N2E4 had the potential application value in the treatment of PDAC, which offered a theoretical basis for targeting therapy of pancreatic cancer.

## Methods

### GEPIA Database

Gene Expression Profiling Interactive Analysis (GEPIA) database is a visualized big data cancer analytics platform based on the cancer genome atlas (TCGA) and the genotype-tissue expression (GTEx). In this study, the difference of neuropilin-2 mRNA (*NRP2*) level between tumor with normal tissues in PDAC, and the relationship between NRP2 levels with the survival prognosis of pancreatic cancer was analyzed by “Expression Plots” and “Survival Plots” modules in the GEPIA database respectively.

### Immunohistochemistry

Tissue arrays (HPan-Ade120Sur-01) were purchased from Shanghai Outdo Biotech Co. Ltd (Shanghai, China), including 63 pairs of pancreatic carcinoma tissues and adjacent para-carcinoma tissues. The antigen retrieval was conducted in a microwave oven with the citrate buffer. Arrays were incubated in endogenous peroxidase blocking solution (reagent A, P003IH, Auragene) for 10 min at room temperature and blocked by normal non-immunized goat serum (reagent B) for 10 min at room temperature. Tissues were incubated with the NRP2 antibody (Affinity, DF7604) at 4°C overnight. Biotin-conjugated (reagent C) secondary antibody and HRP-labeled streptavidin (reagent D) were implemented in turn. After diaminobenzidine (DAB) signals generated, the slides were counterstained with hematoxylin and observed by the micro digital sectioning scanning system (Motic VM1, Hong Kong).

### Evaluation of Immunohistochemical Staining

Immunohistochemistry results were blindly scored by two pathologists: 5 high-power fields were randomly selected from each section. The scores were comprehensively scored according to the intensity of staining and the proportion of positive cells. Staining intensity score: 1 point for the weak positive, 2 points for the medium positive, and 3 points for the strong positive. Positive cell number ratio score: positive cell number <5% is 0, 1%~10% is 1, 11%~50% is 2, 51%~80% is 3, and 81–100% is 4. Results calculation: staining intensity score multiplying positive cell number ratio score (range from 0 to 12). Based on evaluation score, the grade was classified into: 0 point (-); 1~4 points (+); 5~8 points (++); 9~12 points (+++). The cutoff value distinguishing the high and low expression of NRP2 is selected by statistical analysis of the log-rank test: ≤ 8 was categorized as low expression, and >8 was considered as high expression.

### Cell Culture

PDAC cell lines (PANC-1, BxPC-3, MIA PaCa-2) were purchased from the Cell Bank of the Chinese Academy of Sciences (Shanghai, China). Cells were cultured in DMEM medium, or RPMI medium 1640 (Gibco, USA) supplemented with 10% fetal bovine serum (Gibco, USA) and 1% penicillin-streptomycin (Gibco, USA) at 37°C in 5% CO2 humidified atmosphere. All cell lines were authenticated using short tandem repeat, routinely tested for mycoplasma, and passaged in the laboratory for fewer than four months after resuscitation.

### Real-Time qPCR (RT-PCR)

The RNA extraction was implemented using an RNA isolation kit (R401-01, Vazyme), and cDNAs were produced using a HiScript II 1st Strand cDNA synthesis kit (R211-02, Vazyme). SYBR Green (Q711-02, Vazyme) was used as the qPCR master mix. The glyceraldehyde­3­phosphate dehydrogenase (GAPDH) was considered an internal control. The sequences of qPCR primers were acquired from the PrimerBank (https://pga.mgh.harvard.edu/primerbank/) and were as follows:

NRP2-RT-F: 5′- CCAACGGGACCATCGAATCTC -3′
NRP2-RT-R: 5′- CCAGCCAATCGTACTTGCAGT -3′;
VEGF-RT-F: 5′-AGGGCAGAATCATCACGAAGT-3′
VEGF-RT-R: 5′-AGGGTCTCGATTGGATGGCA-3′;
GAPDH-RT-F: 5′- AGAACATTCACGAGTCCTGC -3′
GAPDH-RT-R: 5′- GTGGTCGATGCGGTAGATC -3′.

The relative expression levels of mRNAs were calculated using the 2^-(ΔCt sample–ΔCt control)^ method. Experiments were performed in triplicate.

### Flow Cytometry

To determine whether N2E4 combined with tumor-associated NRP2, flow cytometry was performed. Cells were incubated with either N2E4 (1:100), isotype IgG control, or PBS control for 40 min at 37°C followed by incubation with FITC-conjugated goat anti-mouse IgG (1:20∼1:100, SA00003-1, Proteintech) at 37°C for 40 min. Ten thousand flow cytometry events were collected for each sample with a flow cytometer (Beckman Gallios, USA), and fluorescence emission at 525 nm (FL1) was measured. Data were managed using FlowJo (Tree Star, Ashland, Oregon, USA).

### Western Blotting

Cells treated with N2E4 or PBS were lysed with lysis buffer according to the manufacturer’s protocols (Beyotime Biotechnology). The protein concentration of collected lysates was measured by the BCA protein assay kit (Yeasen Biotech). The equivalent protein sample was added to an equal volume of 2 × SDS loading buffer and boiled for 15 min in boiling water. The protein lysates were separated in the SDS-polyacrylamide gel, followed by being transferred to PVDF membranes (Millipore, Billerica, USA) and blocked using TBST containing 5% skim milk. The PVDF membranes were incubated using primary antibodies specific for NRP2 (Affinity, DF7604), E-cadherin (60335-1-Ig), N-cadherin (22018-1-AP), FAK (Proteintech, 12636-1-AP), p-FAK (CST, 8556), Erk (Abcam, ab196883), p-Erk (CST, 4376), Akt (CST, 2966), p-Akt (CST, 4060), integrinβ1 (Abcam, ab24693), HIF-1α (CST, 36169), β-actin (Abcam, ab8226), or GAPDH (proteintech, 60004-1) at 4°C overnight. HRP-labeled secondary antibody solution (1: 5 000) was added and incubated at room temperature for 1 h. Immunoreactive bands were finally detected using Enhanced Chemiluminescence (ECL) system (5200S, Tanon, Shanghai, China).

### Immunofluorescence

Cells were cultured on specialized culture dishes (Sorfa, 201100) overnight at 37°C and fixed with 4% paraformaldehyde. After blocked by PBST with 2% BSA, slides were incubated using the primary antibody at 4°C overnight, then the fluorochrome-conjugated secondary antibody at 37°C for 1 h in the dark and Hoechst 33258 (B1155, Sigma) at room temperature for 5 min in the dark. The fluorescence images were acquired using a confocal microscope (Olympus FV1000MPE-B, Japan).

### CCK8 Assays

To assess cell proliferation capability, the Cell Counting Kit-8 (CCK8) was obtained from Yeasen Biotech Co., Ltd. (Shanghai, China). Cells were seeded in 96-well plates (2~5 × 10^3^ cells/well) and maintained for 24 h, 48 h, and 72 h with different doses of mAb. CCK8 solution was then added into each well followed by incubation for 2 h. Finally, absorbance was measured at 450 nm using a SPECTRAmax Microplate Reader (model 680, Bio-Rad, Tokyo, Japan). Each experiment was independently repeated three times.

### Scratch Assays

Scratch assay was performed on 90% confluent cells and inoculated in 24-well plates. Scratches were established using sterile 200-μl pipette tips gently. Then cells were cultured with fresh medium containing 400 μg/ml N2E4 or corresponding PBS. When cells migrated into the scratches at 24 h, images of the scratch areas were captured by phase-contrast microscope (Olympus FV1000, Japan) and analyzed by ImageJ software. Each scratch coverage was estimated in quadruplicate at four different locations.

### Matrigel-Coated Invasion Assays

The cell suspension containing 5.0 x 10^4^ cells/ml was prepared in the serum-free medium. Subsequently, 600 μl of complete medium containing 10% fetal bovine serum was added to the lower chamber. 500 μl of cell suspension described above was added to each upper chamber (Corning 3422, USA) to the culture at 37°C for 48 h. Cells below the membranes were stained by 0.5% crystal violet for 20 min at room temperature, washed with water, and air-dried. The numbers of invaded cells were observed and counted under the microscope (Olympus BX53, Japan).

### Immunoprecipitation (IP)/Co-IP

The cells were lysed on ice with 1×lysis buffer containing 1 mM PMSF and centrifuged at 13,000 g for 30 min. The supernatant was then collected. The total protein was incubated with 1 μg NRP2 antibody and 10 μL protein A/G beads overnight at 4°C, C-9 antibody and protein A/G beads as the positive control, IgG and protein A/G beads as the negative control. Wash the protein A/G- beads 3 times. After the last washing, 1× Lysis Buffer was mixed with the same volume of 2×SDS sample buffer and then boiled at 100°C for 10 minutes. The mixture was analyzed by SDS-PAGE electrophoresis. Silver staining or western blotting was performed.

### ELISA

The supernatant culture medium from the co-culture was collected and cytokine levels were measured separately. The supernatant was centrifugated at 12,000 × g for 10 min at 4°C following being collected at 12 h, 24 h, and 48 h. This was then used to measure the VEGF level using a Human VEGF-A Elisa kit (EY-00H100681, EYBIO, Shanghai, China), according to the manufacturer’s protocol.

### Tumor Xenograft Model

All animals were applied under guidelines established by the Animal Care and Use Committee of Xiamen University. Female BALB/c nude mice were gained from the Animal Center of Xiamen University (Fujian, China) and kept in a specific pathogen-free barrier facility indoor. 5 × 10^6^ cells were injected subcutaneously into the back of the right forelimb of 4~6-week-old female BALB/c nude mice. When tumor volume reached 40 ~ 70 mm^3^, estimated as V = L × W^2^/2 (V: Tumor volume; L: Length; W: Width), mice were randomly divided into three groups with 5 animals in each group. Then mice were treated with PBS (vehicle control), low dose (20 mg/kg), and high dose (40 mg/kg) of N2E4 once daily *via* tail vein injection. Tumor volume and body weight were measured three times per week for 2∼3 weeks. After mice were euthanized using CO_2_, tumor tissues were isolated and measured.

### Luciferase Reporter Assay

The BALB/c nude mice were injected about 1 × 10^6^ luciferase-labeled BxPC-3 cells into the lateral tail vein. Mice were anesthetized and injected with D-luciferin potassium salt (E011306, ENERGY, Shanghai, China) for bioluminescent imaging weekly. Images were captured, and the bioluminescent signal was quantified using IVIS Lumina II Imaging System (Caliper, USA). The mice were sacrificed after 8 weeks. The lungs were isolated for hematoxylin and eosin (H&E) staining.

### Statistical Analysis

Statistical analysis was implemented *via* SPSS 17.0 (SPSS Inc, Chicago, IL, USA) and GraphPad Prism8 (GraphPad Software Inc., San Diego, CA, USA) statistical software. Statistical data were expressed as mean ± SD, and the data represent at least three separate experiments. For comparisons of two treatment groups, Student’s t-test was used as appropriate. *P* < 0.05 was seen as statistically significant.

## Results

### Elevated Expression of NRP2 Is Associated With Unfavorable Prognosis in Pancreas Carcinoma

**Figure 1:**
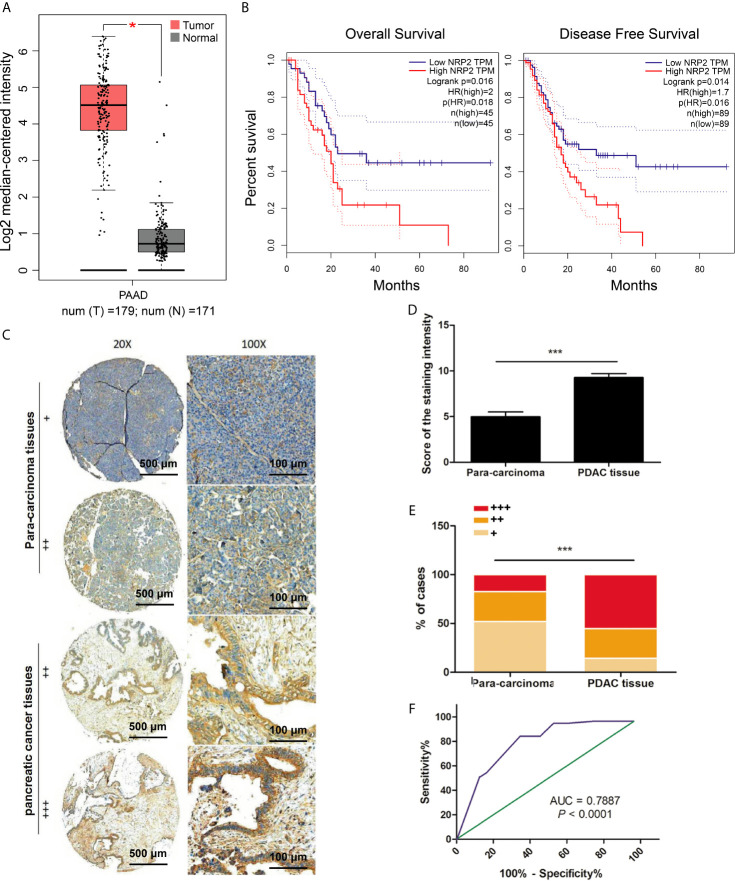
Expression of NRP2 in PDAC. **(A)** Box plots of the nrp2 gene expression in PDAC and normal pancreatic tissues from the GEPIA database, *P < 0.0001. **(B)** The correlation between NRP2 expression with Overall Survival (OS, n=45) or Disease-Free Survival (DFS, n=89) from the GEPIA database, *P* < 0.05. **(C)** NRP2 protein expression level was analyzed by IHC based on a TMA containing 60 pancreatic cancer specimens. Significant differences in **(D)** scores of the staining intensity and **(E)** proportions of 3 cases (strong, +++; moderate, ++; weak, +) between pancreatic cancer and adjacent para-carcinoma tissues, ***P < 0.0001. **(F)** ROC (receiver operating characteristic) curve for NRP2 was plotted by sensitivity and specificity, AUC = 0.7887, 95% CI = 0.7028 ~ 0.8745, *P* < 0.0001.

We assessed the role of *NRP2* in pancreatic carcinoma *via* the GEPIA database. The level of *NRP2* was significantly up-regulated in pancreatic cancer tissues compared with normal pancreatic tissues (*P*< 0.05) (**Figure 1A**). The survival analysis showed that Overall Survival and Disease-Free Survival (**Figure 1B**) of pancreatic cancer patients with high *NRP2* levels were significantly lower than patients with low *NRP2* levels (*p* < 0.05).

Given the above findings, we further analyzed the level of NRP2 in 60 pairs of pancreatic carcinoma tissues and adjacent normal tissues *via* immunohistochemistry. The data confirmed that NRP2 expression was significantly higher in PDAC (**Figure 1C**). The staining intensity score was higher in PDAC tissues than para-carcinoma tissue (P < 0. 0001, **Figure 1D**). Strongly positive (+++) NRP2 expression was confirmed in 55.36% of PDAC tissues and 17.31% of para-carcinoma tissues, weekly positive (+) NRP2 expression was evaluated in only 14.29% of PDAC tissues and 51.92% of para-carcinoma tissues (**Figure 1E**). The ROC curve was created by sensitivity and specificity of NRP2 scores and provided the AUC (0.7887) to distinguish pancreatic cancer from normal tissues (95% CI: 0.7028 to 0.8745; P < 0.0001). The point close to (0.0, 1.0) on the curve maximized sensitivity (84.2%) and specificity (65.5%) (**Figure 1F**). Collectively, the level of NRP2 was significantly raised in pancreatic carcinoma tissues and had higher sensitivity and specificity to differentiate pancreatic cancer from para-carcinoma tissues. Therefore, NRP2 could represent an attractive therapeutic target for PDAC.

### The N2E4 Antibody Specifically Binds to NRP2 in PDAC Cells

**Figure 2:**
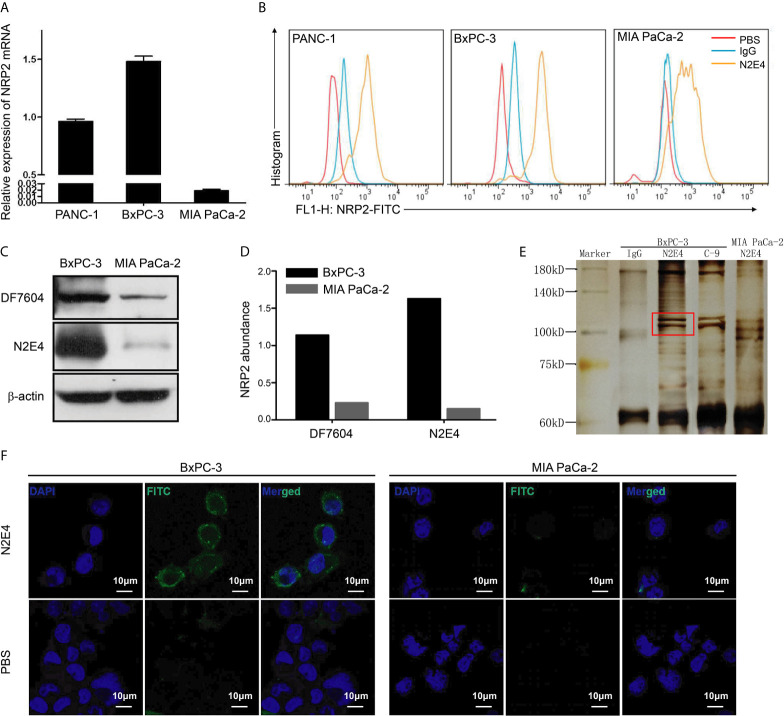
N2E4 combines the membrane-associated NRP2 in PDAC cells. **(A)** Nrp2 gene was detected by RT-PCR in pancreatic ductal adenocarcinoma cell lines PANC-1, BxPC-3, and MIA PaCa-2. **(B)** The binding capability of N2E4 to NRP2 expressed on cells was showed using flow cytometry. **(C)** NRP2 expression and the affinity of N2E4 were further evaluated using western blotting, and **(D)** NRP2 abundance was calculated by Image J. **(E)** After immunoprecipitation by N2E4 antibody, the N2E4-NRP2 complexes were separated by SDS-PAGE gel. C-9 antibody is the positive control, IgG is the negative control. **(F)** The binding of N2E4 directly in BxPC-3 and MIA PaCa-2 using immunofluorescence, representative images of cells obtained at ×1200 magnification was shown.

To detect NRP2 levels in three pancreatic ductal adenocarcinoma cell lines, RT-PCR was conducted. The level of NRP2 mRNA in BxPC-3 cells was the highest, followed by PANC-1 then MIA PaCa-2 (**Figure 2A**). Flow cytometry analysis indicated that the binding capability of N2E4 to NRP2 proteins of cell surface was consistent with NRP2 mRNA levels of three cell lines. The increase of affinity was reflected in a conspicuous fluorescent profile offset in the case of N2E4 (**Figure 2B**). Therefore, BxPC-3 with high NRP2 level and MIA PaCa-2 with low NRP2 level were selected and verified using western blotting. The results showed the levels of NRP2 proteins were much higher in BxPC-3 than MIA PaCa-2, and N2E4 exhibited more significant abundance differences suggesting the affinity of N2E4 could reach the level of the commercial polyclonal antibody H-300 (sc-5542, Santa) (**Figures 2C, D**). After immunoprecipitation, the N2E4-protein complex was precipitated by protein A/G beads and then separated on an SDS-PAGE gel. A specific band of around 110 kDa was detected in PDAC cells compared with the IgG control and commercial NRP2 monoclonal antibody C-9 (sc-13117, Santa), which proved that the N2E4 target protein in PDAC cells was NRP2 (**Figure 2E**). Immunofluorescence assay displayed the binding of N2E4 with BxPC-3 cells with strong FITC-fluorescence intensity (**Figure 2F**). Thus, the N2E4 antibody was specifically combined with NRP2 in PDAC cells.

### N2E4 Inhibits Proliferation, Migration, and Invasion in BxPC-3 Cells

**Figure 3:**
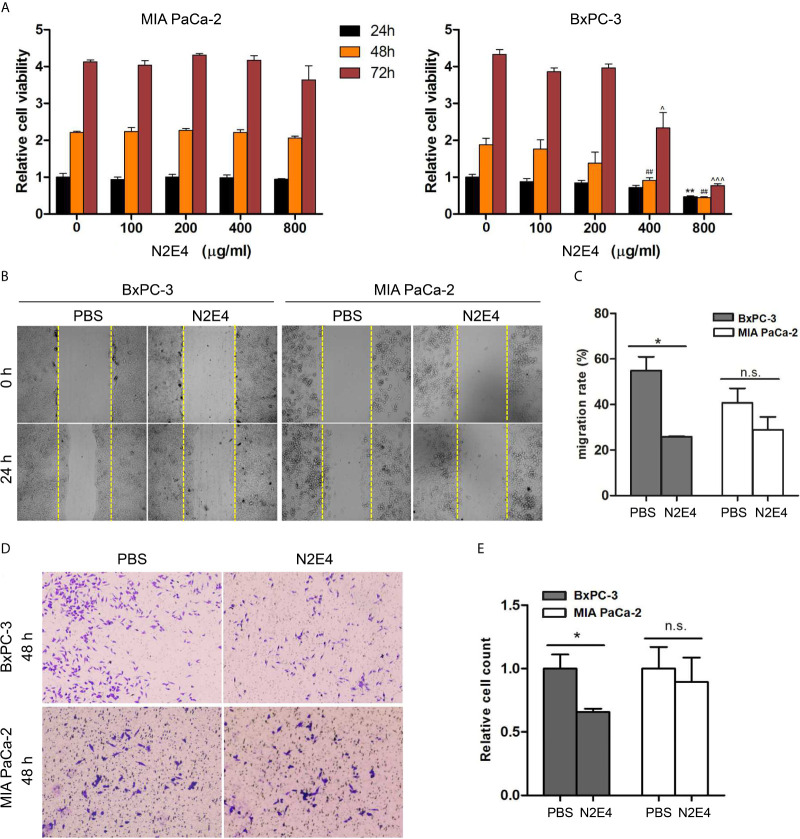
Effect of N2E4 on cell viability, proliferation, invasion, and migration. **(A)** CCK8 assays were performed to compared cell viability against N2E4 in BxPC3 and MIA PaCa-2. Statistical analysis is based on the mean ± SD of at least three independent tests. **P < 0.01 *vs* 0 μg/ml for 24 h group; ^##^ *P* < 0.01 *vs* 0 μg/ml for 48 h group; ^*P* < 0.05, ^^^*P* < 0.001 *vs* 0 μg/ml for 72 h group. **(B)** Scratch assays verified the decrease of migration in BxPC-3 cells after the treatment of N2E4. **(C)** Statistical results were obtained as described above. **(D)** Matrigel-coated assays displayed that N2E4 decreased cell invasion ability in BxPC3. **(E)** Statistical results were obtained as described above. *P < 0.05; **P < 0.01; n.s., no significance.

To determine the effects of the N2E4 antibody on PDAC cells (MIA PaCa-2 and BxPC-3), cell viability, migration, and invasion were measured using cell biology assays. CCK-8 assay displayed that the cell viability of MIA PaCa-2 was hardly changed, but there was slightly decreased after 48 and 72 h of N2E4 treatment (400 μg/ml) in BxPC-3 cell lines (**Figure 3A**). The IC50 values of BxPC-3 cells for N2E4 were calculated using GraphPad and were 698.1 μg/ml, 426.9 μg/ml, and 399.9 μg/ml at 24h, 48h, and 72h, respectively. The results of scratch assays presented that the migration capacity of BxPC-3 cells was significantly inhibited after 24 h of N2E4 treatment compared with the PBS group (**Figures 3B, C**). Similarly, the invasion capacity of BxPC-3 cells was visibly repressed after 48 h of N2E4 treatment using Matrigel-coated assays (**Figures 3D, E**). These data demonstrated that N2E4 contributed to the inhibitions of proliferation, migration, and invasion in BxPC-3 cells.

### N2E4 Affects the Formation of Pseudopods and the E-Cadherin/N-Cadherin Switch in EMT

**Figure 4:**
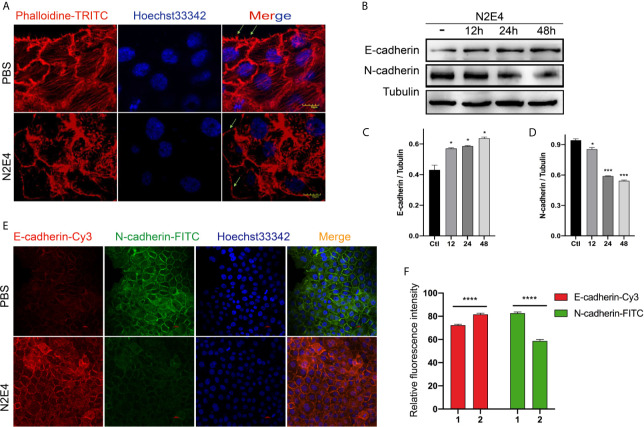
The formation of pseudopods and the expression of E-cadherin/N-cadherin in BxPC-3 cells. **(A)** Phalloidine staining in BxPC-3 with the treatment of PBS or N2E4. Note the intense staining at the leading edge of invasive pseudopod protrusions, marked by arrows. Scale bars = 10 µm. **(B)** The protein levels of E-cadherin and N-cadherin with 12, 24, or 48 h pre-incubation using N2E4 or PBS (-) as measured by western blotting analysis. Densitometric analysis on the levels of **(C)** E-cadherin and **(D)** N-cadherin were normalized against the corresponding tubulin protein levels, which was then designated as 1. Bars are the means ± SD, n = 3. *P < 0.05; ***P < 0.001. **(E)** After pre-incubation using N2E4 or PBS for 48 h, E-cadherin (Cy3, red) and N-cadherin (FITC, green) were measured by immunofluorescence analysis. **(F)** Relative fluorescence intensity of E-cadherin-Cy3 and N-cadherin-FITC was represented. Bars are the means ± SD, n = 3. ****P < 0.0001.

PDAC is characterized by aggressive migration and invasion (18, 19). The increased pseudopods caused the aggressive movement of cancer cells. Lee et al. found formations of pseudopods and stress fibers contributed to cancer cell migratory and invasive ability and tumor metastasis in orthotopic lung cancer animal models (20). In this work, we observed strong skeletal structures, abundant stress fibers, and filamentous pseudopodia (marked with green arrows) in the PBS group *via* rhodamine-labeled phalloidin. In contrast, the N2E4 treatment group showed minimal pseudofoot protrusions, blurred skeleton structures, and reduced stress fibers (**Figure 4A**).

Besides, EMT is the initial cause of tumor invasion and metastasis, one of which is characterized by the loss of epithelial markers (e.g. E-cadherin) and the gain of mesenchymal markers (e.g. N-cadherin) (21, 22). To demonstrate whether N2E4 referred to the regulation of EMT, western blotting and cellular immunofluorescence assays were performed. Compared with the PBS group, the N-cadherin level decreased gradually over time, while the level of E-cadherin increased slightly under the N2E4 treatment (**Figures 4B–D**). And the results of the immunofluorescence (**Figures 4E, F**) were consistent with those of Western blotting analysis. These data supported that N2E4 inhibited the formation of pseudopods and EMT processes in PDAC cells.

### N2E4 Attenuates the Activation of FAK/Erk/HIF-1α by Blocking the Interaction Between NRP2 With Integrinβ1

**Figure 5:**
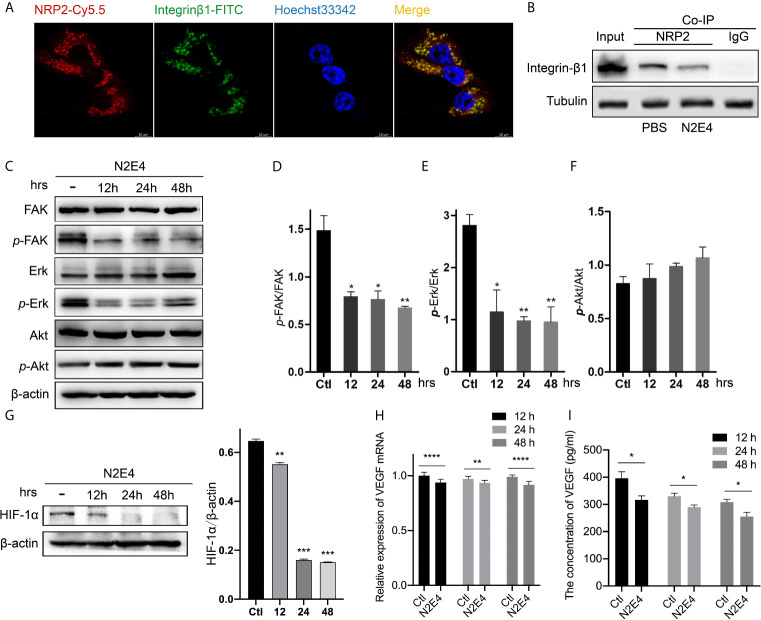
Effect of N2E4 on selected signaling molecules in BxPC-3. **(A)** The co-localization of NRP2-Cy5.5 (red) with integrinβ1-FITC (green). **(B)** After BxPC-3 cells were pre-incubated with PBS (-) or N2E4 for 48 h, NRP2 and integrinβ1 proteins were immunoprecipitated with an anti‐NRP2 antibody and then immunoblotted using an anti‐integrinβ1 antibody. **(C)** After BxPC-3 cells were pre-incubated with N2E4 or PBS (as control) for 12, 24, or 48 h, western blotting was applied to determine levels of the total FAK, Erk1/2, and Akt, also the corresponding phosphorylated proteins. Densitometric analysis of phosphorylated protein levels was standardized against corresponding total protein levels: **(D)** FAK, **(E)** Erk1/2, and **(F)** Akt. **(G)** The test of HIF-1α protein level by western blotting. **(H)** *VEGF* gene was detected by RT-PCR after cells were pre-incubated with N2E4 or PBS for 12, 24, or 48 h. **(I)** Autocrine protein VEGF was detected by ELISA. Bars are the means ± SD, n = 3. *P < 0.05; **P < 0.01; ***P < 0.001; ****P < 0.0001.

To detect whether N2E4 inhibits the interaction between NRP2 and integrinβ1, we verified the co-localization of NRP2 and integrinβ1 using immunofluorescence and detected the interaction intensity between them using Co-IP. As was depicted in **Figure 5A**, significant co-localization of NRP2 with integrinβ1 was exhibited in BxPC-3 cells. The reduced interaction between NRP2 and integrinβ1 under the treatment of N2E4 was further confirmed by Co-IP (**Figure 5B**). Moreover, western blotting was proceeded to examine the changes in protein levels after N2E4 treatment in BxPC-3 cells. We found that phosphorylated FAK and Erk1/2 remarkably decreased with the prolonged action of N2E4, while total FAK, Erk1/2, or Akt and phosphorylated Akt level remained stable (**Figures 5C–F**). It prompted that the activation of FAK and Erk1/2 was suppressed with the treatment of N2E4.

As Erk is involved in the regulation of synthesis and transcriptional activation of HIF-1α (23), we hypothesized that N2E4 might have a role in the transcription and expression of VEGF. So western blotting was firstly performed to analyze whether or not N2E4 was able to affect the expression of HIF-1α. Treatment with N2E4 could effectively reduce the level of HIF-1α protein (**Figure 5G**). Secondly, we analyzed the VEGF mRNA expression level by RT-PCR and the autocrine VEGF protein level using ELISA. The data indicated the levels of both VEGF mRNA (**Figure 5H**) and autocrine protein (**Figure 5I**) were inhibited after N2E4 treatment. The above results indicated that N2E4 might attenuate the activation of FAK/Erk/HIF-1α by blocking the interaction between NRP2 with integrinβ1, then inhibit VEGF signal transduction.

### N2E4 Inhibits the Growth and Metastasis of Pancreatic Cancer *In Vivo*

**Figure 6:**
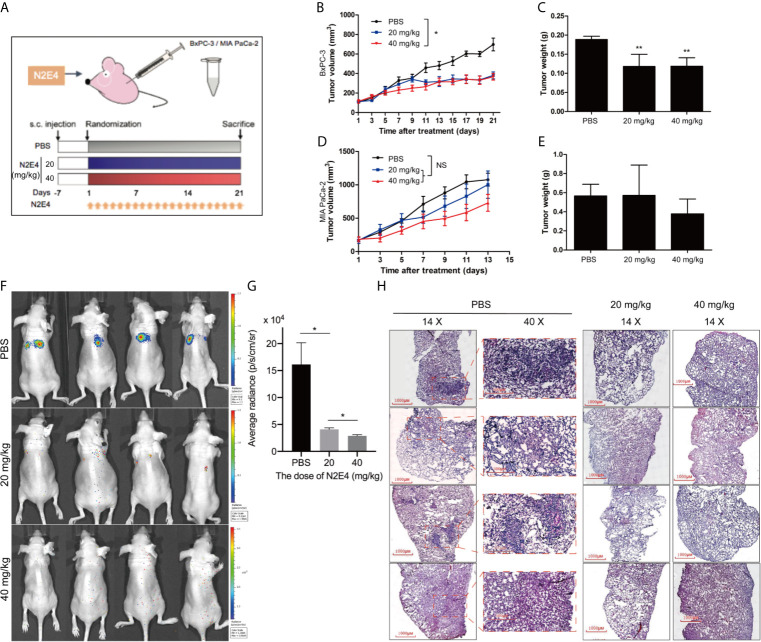
Antitumor effects of N2E4 on pancreatic cancer xenografts. **(A)** BxPC-3 and MIA PaCa-2 cells (5 x 10^6^) were subcutaneously injected into 4~6-week-old female BALB/c nude mice. When tumor volume reached 40 ~ 70 mm^3^, mice were randomly divided into different groups and treated with PBS, low dose (20 mg/kg), and high dose (40 mg/kg) of N2E4 once daily *via* tail vein injection. **(B, D)** Tumor volume curves. **(C, E)** The average weight of the isolated tumor tissues in each group. **(F, G)** Representative images and quantitative analysis of lung metastasis were determined by luciferase-based bioluminescence imaging. **(H)** Metastasis nodules from isolated lungs were evaluated by tissue section H&E staining. *P < 0.05; **P < 0.01. NS, no significance.

All animals were applied under guidelines established by the Animal Care and Use Committee of Xiamen University. To evaluate the effect of N2E4 on the growth of pancreatic cancer *in vivo*, nude mice bearing MIA PaCa-2 and BxPC-3 xenografts were treated with different doses of N2E4 once per day by tail vein injection (**Figure 6A**). And the results showed that N2E4 prompted a more significant growth inhibition effect on BxPC-3 xenograft. The inhibition effect for tumor volume was more evident with a high dose (40 mg/kg) of N2E4, P < 0.05 (**Figure 6B**). In terms of tumor weight, two treatment groups showed significant inhibitory effects (P < 0.01) with a nearly 40% inhibitory rate, however, the two doses did not have different effects (**Figure 6C**). According to Burvenich et al., the receptor occupancy to the antibody-drug *in vivo* correlates with antitumor efficacy, and the saturation of receptors corresponds to maximal antitumor efficacy *in vivo* (24), Therefore, we speculated that the antibody dose of 20 mg/kg might approach the saturation state of NRP2 receptors. By contrast, it was not statistically significant for inhibition effect in tumor growth-mediated by MIA PaCa-2 (**Figures 6D, E**). These results indicated that N2E4 suppressed the NRP2-associated growth of pancreatic cancer cells *in vivo*, consistent with the results *in vitro*. All the mice were well tolerated with stable body weight and without obvious toxicity signs during the study period.

Furthermore, a xenograft nude mouse model was established to further explore whether N2E4 could restrain metastasis of PDAC *in vivo*. The BxPC-3 cells labeled luciferase were injected into the tail vein of nude mice to simulate the spread of cancer cells throughout the blood. All mice survived until the end of the experiment. The heightened metastasis was observed in mice without N2E4 treatment; however, metastasis was markedly inhibited in mice from the N2E4 treatment group (**Figure 6F**). The average radiance of lungs in the N2E4 treatment group was lower compared with the control (P < 0.05), indicating decreased metastasis (**Figure 6G**). Also, the number of lung metastasis nodules was significantly reduced in the N2E4 treatment group using H&E (**Figure 6H**). The data indicated that N2E4 attenuated the metastasis of pancreatic cancer cells.

## Discussion

In this study, the expression of NRP2 is elevated and associated with unfavorable prognosis in pancreas carcinoma (**Figure 1**). Based on our previous report, we further confirm that the N2E4 monoclonal antibody specifically combines with the NRP2 in PDAC cells (**Figure 2**). Our findings suggest that N2E4 inhibits proliferation, migration, invasion (**Figure 3**), and represses growth and metastasis of pancreatic cancer cells *in vivo* (**Figure 6**).

In the migration of pancreatic cancer, cells are often powered by filament pseudopodia involved in the extension in the direction of cell movement (25–27). Ouyang et al. revealed that docetaxel could attenuate the migration and invasion of breast cancer cells compared with doxorubicin, which was associated with suppression of filopodia formation by these agents (28). Therefore, through the specific fluorescence staining of the cytoskeleton, it is clear that with N2E4 treatment, skeletal structures under the cell membrane become blurred, filamentous pseudopods and stress fibers absent gradually (**Figure 4A**), which could mean the aggressive movement and EMT weakens (22). Besides, the increased epithelial phenotype markers (E-cadherin) and the decreased mesenchymal phenotype marker (N-cadherin) under N2E4 treatment further confirmed our hypothesis (**Figures 4B–F**).

The change in cell stiffness determined by the cytoskeleton is a crucial feature of cancer cells that affects the metastatic ability. Zou et al. demonstrated that actin cytoskeleton remodeling led to decreased cell stiffness *via* integrin/FAK/Erk Pathways, enhancing the migration of mesenchymal stem cells (29). Also, NRP2 is necessary for the association of α6β1-integrin with the cytoskeleton and also facilitates integrinα6β1-mediated activation of FAK (10). We demonstrated that N2E4 inhibited the activation of FAK/Erk by blocking the interaction between NRP2 with integrinβ1 in BxPC-3 cells using Co-IP and western blotting, but Akt signaling inhibition is not involved in the mechanism (**Figures 5A–F**). Erk is not only involved in the regulation of HIF-1α synthesis but also its transcriptional activation. The phosphorylated Erk could increase the transcriptional activation function of HIF-1α, and thus stimulates the biological function of VEGF (23), for instance, survival and metastasis of pancreatic cancer (16). Interestingly, our data revealed that the expression of HIF-1α was attenuated with the N2E4 treatment (**Figure 5G**), and VEGF mediated signal transduction might be further inhibited (**Figures 5H, I**).

One limitation of our research is that pancreatic cancer characteristically contains dense stroma in the tumor tissue, which might affect the drug response and resistance. However, mimicking the dense stroma by xenograft tumor is relatively complicated, and the genetically-engineered mouse model might have an advantage on this issue. Besides, for the treatment of PDAC with highly therapy-resistant (30), it is crucial to determine which patients will benefit most from an approach and tailor precise drugs and treatments to individual patients (31). For example, the PD-L1 expression level has been accepted as the companion diagnostic (for lung cancer) or the complementary detection (for melanoma and bladder cancer) on anti-PD-1 treatments (32). As the treatment requires a direct interaction between NRP2 and N2E4, N2E4 shows more practical effects on pancreatic cancer cell lines and xenografts with high NRP2 expression. Therefore, the expression of the receptor and its assessment would constitute vital steps for clinical application, which is likely significant for the development of precision medicine.

**Figure 7:**
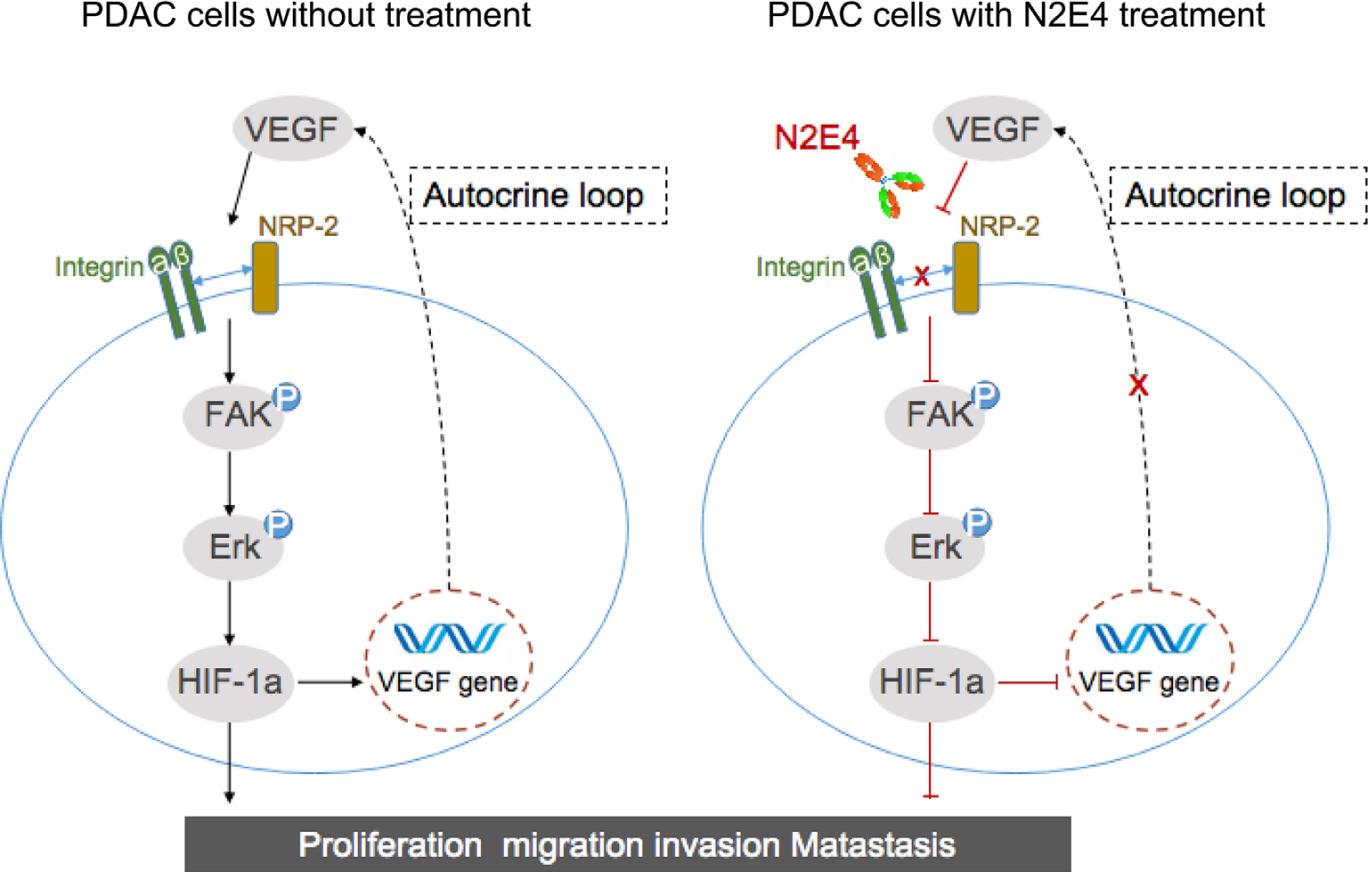
Schematic presentation of potential mechanisms of N2E4 antibody in pancreatic cancer cells. N2E4 weakens the interaction between NRP2 and integrinβ1 by blocking NRP2, thereby inhibiting the cascade reaction of FAK/Erk/HIF-1α, then inhibit autocrine VEGF signal transduction. This directly restrains the proliferation, migration, invasion, and metastasis, to impede the development of pancreatic cancer.

Although this is merely the tip of the icebergs in assessing the application potential of NRP2 pathway blockade by N2E4, these findings suggested that N2E4 could restrain interaction between NRP2 with integrinβ1 to inhibit FAK/Erk/HIF-1a/VEGF signaling, limiting growth and metastasis of pancreatic cancer (**Figure 7**). Actually, antibody drugs have two main physiological activities *in vivo*: one is to recognize and specifically bind antigen substances through the Fab segment, causing the neutralization or apoptosis of the antigen. The second is the immunological effect of antibodies, for instance, antibody-dependent cell-mediated cytotoxicity (ADCC). The Fc segment of the antibody has effector cell ligand binding site and can bind to FcγRIIIA on the surface of NK cells (effector cells) to mediate the ADCC action (33). It is worth mentioning that Liu et al. found that NRP1 determines how T cells develop and establish immune memory, and knocking out NRP1 can enhance the immune memory ability of T cells, and the immune response is more robust when the tumor cells are “seen” again (34). Therefore, the role of NRP2, which is highly homologous to NRP1, in memory immunity may be worth looking forward to. Or maybe the inhibitory effect of N2E4 on pancreatic cancer is also associated with enhanced immune response.

In conclusion, as a unique therapeutic monoclonal antibody inhibiting the crucial signaling pathway, N2E4 suppresses PDAC cell proliferation, migration, invasion *in vitro*, and significantly inhibited tumor growth and metastasis *in vivo*. Consequently, this work suggests that N2E4 has the potential for targeting therapy of PDAC, contributes to understanding the NRP2 function in cellular movement and tumor metastasis, and lays a foundation for the future development of NRP2-based targeted therapy for PDAC.

## Data Availability Statement

The raw data supporting the conclusions of this article will be made available by the authors, without undue reservation.

## Ethics Statement

The animal study was reviewed and approved by Animal Care and Use Committee of Xiamen University.

## Author Contributions

LW, JY, FL, and TW conceived the idea. LW designed research. LW and LLW performed experiments. SW and ZZ purified antibodies. LW, PX, and XL collected data and interpreted the results. LW and ZL wrote the paper. All authors contributed to the article and approved the submitted version.

## Funding

The work is funded by the National Natural Science Foundation of China with grant number 81773770, and the Provincial Public Welfare Project of Fujian Province, China with the grant number 2018R1036-1, 2018R1036-3, 2019R1001-2, and 2020R1001001.

## Conflict of Interest

The authors declare that the research was conducted in the absence of any commercial or financial relationships that could be construed as a potential conflict of interest.
